# Complete genome sequence of *Paramuribaculum intestinale* DSM 100749^T^, isolated from feces of C57BL/6 laboratory mice

**DOI:** 10.1128/MRA.00797-23

**Published:** 2023-10-31

**Authors:** Adrian Low, Xiu Qi Koh, Yichen Ding, Henning Seedorf

**Affiliations:** 1 Temasek Life Sciences Laboratory, Research Link, Singapore, Singapore; 2 Department of Biological Sciences, National University of Singapore, Singapore, Singapore; Department of Biological Sciences, Wellesley College, Wellesley, Massachusetts, USA

**Keywords:** genomes, anaerobes, gut microbiome, Muribaculaceae

## Abstract

Here, the complete genome of *Paramuribaculum intestinale* type strain DSM 100749^T^(=JCM 33114^T^) is presented. *P. intestinale* is a recently described species of *Muribaculaceae* and was isolated from the gut of C57BL/6 laboratory mice. The genome can serve as an important resource for comparative genomics approaches.

## ANNOUNCEMENT

Bacterial species belonging to *Muribaculaceae* are prevalent in the mouse intestine (1 – 3). However, few genomes of *Muribaculaceae* isolates have been completely sequenced (4 – 8). *Paramuribaculum intestinale* DSM 100749^T^(=JCM 33114^T^) was recently isolated with only a draft genome available (378 contigs; RefSeq:GCF_003024925.1) (1, 9). *P. intestinale* cells are catalase-positive, Gram-negative short rods (4). *P. intestinale *showed an increase in relative abundance when male C57BL/6 mice matured (10). *P. intestinale* may be more resilient to dietary change from standard chow to a high-fat/sugar diet compared to other *Muribaculaceae* species (11). An *in vivo* study of a gavage microbiota including *P. intestinale* appeared to suppress murine hosts’ cravings for high-sucrose pellets (12). Hence, having the complete genome of *P. intestinale* could benefit researchers utilizing C57BL/6 mice for research.

Strain DSM 100749^T^ was acquired from a German culture repository (DSMZ) as an active culture in chopped-meat broth and was sub-cultured 10% (vol/vol) in 4 mL anaerobic Mega broth using a recipe previously described (13). Culturing and incubation were performed in a Coy anaerobic chamber. DNA was extracted from a 4 mL culture incubated at 37°C for 72 h. DNA extraction followed the protocol for the PowerFecal Pro kit (Qiagen) except where the bead-beating period was reduced to 6 min at maximum speed on a Vortex-Genie 2 and further purified using Genomic-tip 20/G (Qiagen) with DNA buffer set (Qiagen) without modifications to the manufacturer’s protocol. The genomic library for 150 bp paired-end sequencing was prepared using the NEBNext Ultra II FS DNA Library Prep Kit (New England Biolabs) and sequenced on a NovaSeq 6000 sequencer (Illumina) according to the manufacturer’s protocol. Default parameters for all software were applied unless stated otherwise. Raw reads were quality filtered using fastp v.0.23.2 to remove adapter sequences and trimmed (quality score ≥Q5, ambiguous bases = 50%, N_basenumber = 15) generating 9,282,568 filtered reads (1.39 Gbp) (14). A MinION sequencer attached to a FLO-MIN106 flow cell from Oxford Nanopore Technologies (ONT) was used to sequence the DNA library prepared using the SQK-LSK109 ligation sequencing kit (ONT) without DNA shearing or size selection. ONT raw reads were base-called, and quality filtered (quality score ≤7) using a MinIT (ONT; Guppy v3.2.10) resulting in 75,908 (576 Mbp, N50 = 10.60 kb) filtered reads (15).

A single circular replicon of 3,056,428 bp (GC content = 52.5%) orientated with *dnaA* as the forward strand was constructed by short- (×456 coverage) and long-read (576.8 Mbp, ×191 coverage) assembly using UniCycler v.0.4.8 (16). Circularization (0% gap) and genome quality (99.43% completeness, 0.94% contamination) were verified using BUSCO v.5.4.7 and CheckM v.1.2.2, respectively (17, 18). NCBI Prokaryotic Genome Annotation Pipeline v.6.1 revealed 2,729 genes (2,618 coding), and 2,657 coding sequences of which 2,618 are encoded for proteins and 39 pseudogenes (19). RNAs consisted of 4 complete 5S, 16S, and 23S each, 57 tRNA, and 3 ncRNAs. There is one CRISPR array. The sequenced genome increased the reference genome (RefSeq:GCF_003024815.1) by 127,385 bp with 99.76% average nucleotide identity (93.2% alignment) using the BLAST algorithm (20). We hereby share the complete genome of *P. intestinale* DSM 100749^T^.

## Data Availability

The raw Illumina and MinION reads were deposited to NCBI SRA with SRR19725954 and SRR19725955 as accession numbers, respectively. The complete genome is accessible by https://www.ncbi.nlm.nih.gov/nuccore/CP098825.1/ with CP098825.1 as GenBank accession number.
